# Supplementary material for: Enzymes with Lactonase Activity against Fungal Quorum Molecules as Effective Antifungals
Source: Biomolecules. 2024 Mar 21;14(3):383. doi: 10.3390/biom14030383 (PMC10968368; doi:10.3390/biom14030383)
Supplement: Supplementary file 1 [file biomolecules-14-00383-s001.zip › biomolecules-2897888-supplementary.pdf]

Supplementary Materials

Biomolecules

Enzymes exhibiting lactonase activity against fungal QS molecules as effective antifungals

Elena Efremenko*, Aysel Aslanli, Maksim Domnin, Nikolay Stepanov and Olga Senko

Faculty of Chemistry, Lomonosov Moscow State University, Lenin Hills 1/3, Moscow 119991, Russia
*Correspondence: elena_efremenko@list.ru; Tel.: +7-(495)-939-3170

TableS1. Values of affinity and area occupied by lactone containing signaling molecules of fungal QS on the surface of enzymes, exhibiting lactonase activity.

Enzyme	Quorum molecule	Occupied area, %		Affinity, kJ·mol^{-1}
		Near active sites	Total	
AaL	γ-butyrolactone	40.6	4.5	-14.6 ± 0.8
	γ-heptalactone	61.2	4.6	-20.3 ± 1.4
	Butyrolactone I	68.6	5.2	-31.6 ± 1.2
	Multicolanic acid	72.5	3.6	-24.1 ± 0.7
	Multicolic acid	61.0	8.3	-23.0 ± 0.9
	Multicolosic acid	68.3	5.9	-23.6 ± 2.1
AidC	γ-butyrolactone	59.2	2	-15.5 ± 0.7
	γ-heptalactone	0	2.9	-19.7 ± 0.4
	Butyrolactone I	35.4	7.5	-34.5 ± 3.2
	Multicolanic acid	0	3.7	-24.1 ± 1.0
	Multicolic acid	34.4	3.5	-26.8 ± 0.1
	Multicolosic acid	0	4.0	-27.0 ± 1.7
AiiA	γ-butyrolactone	42.1	5.2	-13.2 ± 1.1
	γ-heptalactone	41.7	6.9	-17.8 ± 0.8
	Butyrolactone I	4.0	13.3	-28.2 ± 2.3
	Multicolanic acid	7.3	7.0	-21.8 ± 1.6
	Multicolic acid	4.0	6.5	-20.9 ± 1.4
	Multicolosic acid	7.3	4.9	-20.7 ± 1.5
AiiB	γ-butyrolactone	1.1	1.6	-15.9 ± 0.8
	γ-heptalactone	4.5	2.1	-19.5 ± 1.1
	Butyrolactone I	4.5	3.2	-36.4 ± 1.4
	Multicolanic acid	18.6	5.1	-23.4 ± 1.1
	Multicolic acid	0	2.3	-28.7 ± 1.0
	Multicolosic acid	16.1	5.1	-23.4 ± 1.4
His_6-OPH	γ-butyrolactone	77.0	2.2	-16.7 ± 0.4
	γ-heptalactone	42.4	1.8	-21.7 ± 1

	Butyrolactone I	15.2	6.9	-31.8 ± 1.6
	Multicolanic acid	7.2	5.4	-23.6 ± 1.2
	Multicolic acid	14.1	5	-22.2 ± 0.9
	Multicolosic acid	11.5	5.6	-23.4 ± 0.9
ZEN	γ-butyrolactone	83.8	2.3	-15.1 ± 0.7
	γ-heptalactone	97.7	3.5	-18 ± 2.3
	Butyrolactone I	0	14.3	-26.6 ± 1.1
	Multicolanic acid	97.7	6.6	-22.6 ± 2.3
	Multicolic acid	97.7	7.6	-22.6 ± 2
	Multicolosic acid	94.0	10.4	-19.9 ± 1.5
SsoPox	γ-butyrolactone	50.4	2.6	-16.3 ± 0.9
	γ-heptalactone	52.4	4.7	-21.5 ± 0.7
	Butyrolactone I	12.6	8.7	-34.3 ± 5.1
	Multicolanic acid	0	2.1	-27.8 ± 0.7
	Multicolic acid	27.5	3.2	-26.8 ± 0.9
	Multicolosic acid	20.8	2.7	-25.9 ± 1.3
PvdQ	γ-butyrolactone	14.1	2.2	-16.7 ± 1
	γ-heptalactone	35.9	3.3	-19 ± 1.2
	Butyrolactone I	94.5	5.1	-31.0 ± 0.7
	Multicolanic acid	57.1	4.4	-22.6 ± 0.4
	Multicolic acid	67.9	4.7	-24.1 ± 3.0
	Multicolosic acid	82.0	3.5	-19.5 ± 1.1
MiM-1	γ-butyrolactone	77.5	8.2	-13.6 ± 0.8
	γ-heptalactone	76.7	10.9	-15.9 ± 0.9
	Butyrolactone I	94.7	16.1	-27 ± 0.7
	Multicolanic acid	87.5	14.7	-19.7 ± 1.1
	Multicolic acid	91.8	16.2	-20.7 ± 1.1
	Multicolosic acid	91.9	17.9	-18.4 ± 0.7
MIM-2	γ-butyrolactone	16.1	4.1	-13.8 ± 0.3
	γ-heptalactone	66.1	3.7	-16.5 ± 1.4
	Butyrolactone I	53.1	7.6	-29.5 ± 1.1
	Multicolanic acid	90.4	8.1	-21.3 ± 1.1
	Multicolic acid	84.0	7.9	-21.1 ± 1
	Multicolosic acid	80.7	10.3	-19.7 ± 1.4
NDM-1	γ-butyrolactone	0	1.8	-16.7 ± 1.1
	γ-heptalactone	10	3.1	-24.3 ± 2.9
	Butyrolactone I	54.6	7.1	-28 ± 0.7
	Multicolanic acid	61.4	4.9	-22.5 ± 3.1
	Multicolic acid	56.9	7.8	-21.7 ± 0.6
	Multicolosic acid	24.7	6.5	-19.7 ± 0.2

Table S2. Theoretically calculated K_m values for His$_6$-OPH in the reactions of hydrolysis of lactone containing signaling molecules of fungal QS.

Quorum molecule	K_m, μM
γ-butyrolactone	1018.6
γ-heptalactone	315.8
Butyrolactone I	29.6
Multicolanic acid	202.4
Multicolic acid	280.9
Multicolosic acid	212.1

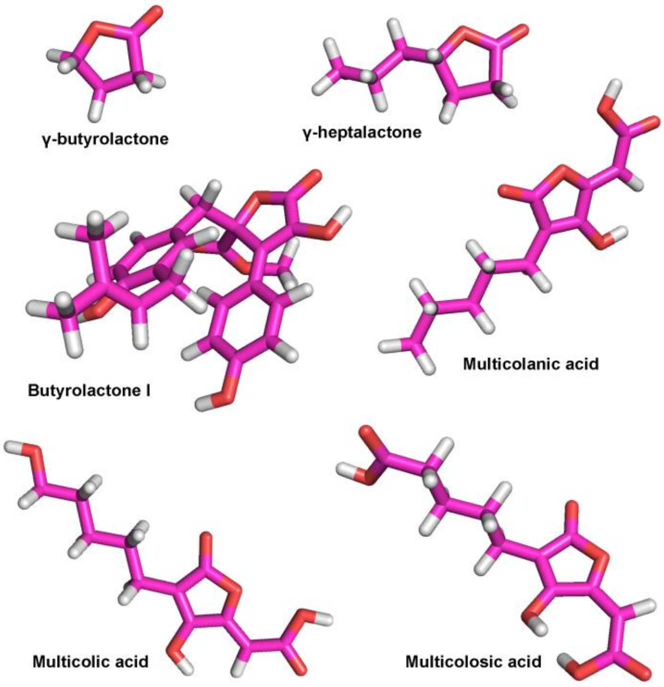

Figure S1. Three-dimensional structure of lactone-containing molecules of fungal QS used in molecular docking simulations.

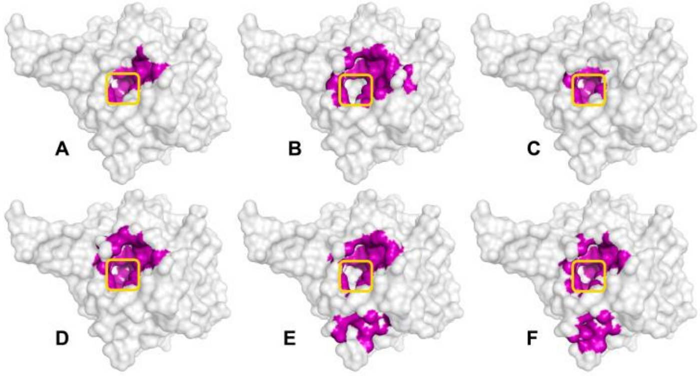

Figure S2. Localization of signaling molecules of fungal QS (γ-butyrolactone (A), butyrolactone I (B), γ-heptalactone (C), multicolanic acid (D), multicolic acid (E) and multicolosic acid (F)) on the surface of **AaL** enzyme, exhibiting lactonase activity. Molecular surface of enzymes, shown as translucent, colored gray. The atoms located within 4 Å of any QS molecule atom and the corresponding molecular surface of enzyme are colored purple. The entrances to the active sites of enzymes are highlighted with yellow boxes.

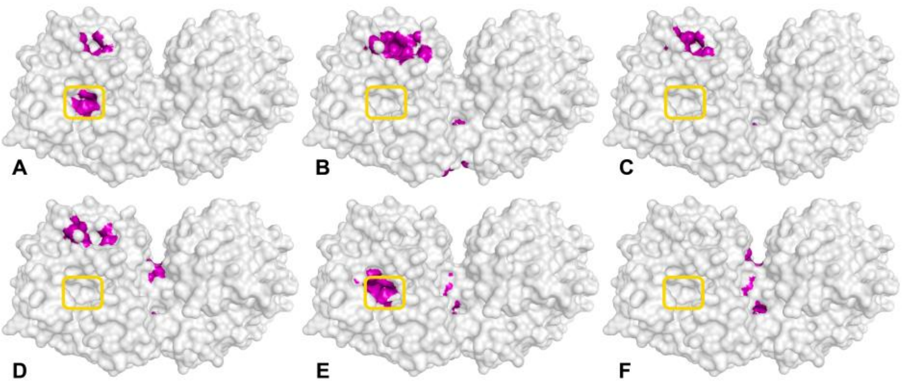

Figure S3. Localization of signaling molecules of fungal QS (γ-butyrolactone (A), butyrolactone I (B),γ-heptalactone (C), multicolanic acid (D), multicolic acid (E) and multicolosic acid (E)) on the surface of **AidC** enzyme, exhibiting lactonase activity. Molecular surface of enzymes shown as translucent colored gray. The atoms located within 4 Å of any QS molecule atom and the corresponding molecular surface of enzyme, are colored purple. The entrances to the active sites of enzymes are highlighted with yellow boxes.

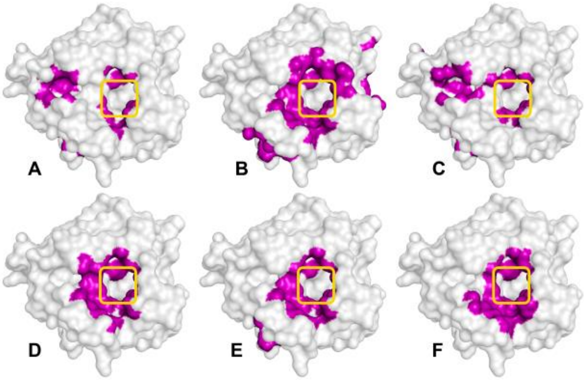

Figure S4. Localization of signaling molecules of fungal QS (γ-butyrolactone (A), butyrolactone I (B),γ-heptalactone (C), multicolanic acid (D), multicolic acid (E) and multicolosic acid (E)) on the surface of **AiiA** enzyme, exhibiting lactonase activity. Molecular surface of enzymes shown as translucent colored gray. The atoms located within 4 Å of any QS molecule atom and the corresponding molecular surface of enzyme, are colored purple. The entrances to the active sites of enzymes are highlighted with yellow boxes.

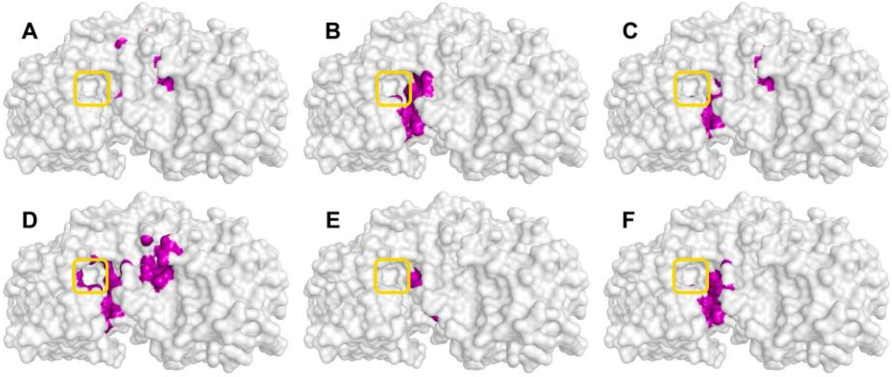

Figure S5. Localization of signaling molecules of fungal QS (γ-butyrolactone (A), butyrolactone I (B),γ-heptalactone (C), multicolanic acid (D), multicolic acid (E) and multicolosic acid (E)) on the surface of **AiiB** enzyme, exhibiting lactonase activity. Molecular surface of enzymes shown as translucent colored gray. The atoms located within 4 Å of any QS molecule atom and the corresponding molecular surface of enzyme, are colored purple. The entrances to the active sites of enzymes are highlighted with yellow boxes.

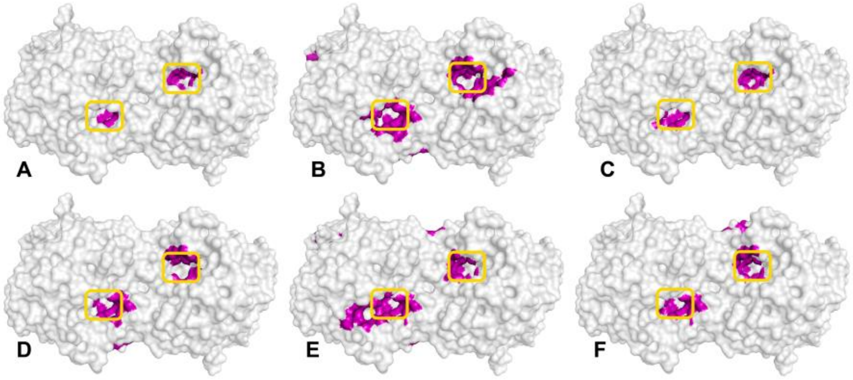

Figure S6. Localization of signaling molecules of fungal QS (γ-butyrolactone (A), butyrolactone I (B),γ-heptalactone (C), multicolanic acid (D), multicolic acid (E) and multicolosic acid (E)) on the surface of **His₆-OPH** enzyme, exhibiting lactonase activity. Molecular surface of enzymes shown as translucent colored gray. The atoms located within 4 Å of any QS molecule atom and the corresponding molecular surface of enzyme, are colored purple. The entrances to the active sites of enzymes are highlighted with yellow boxes.

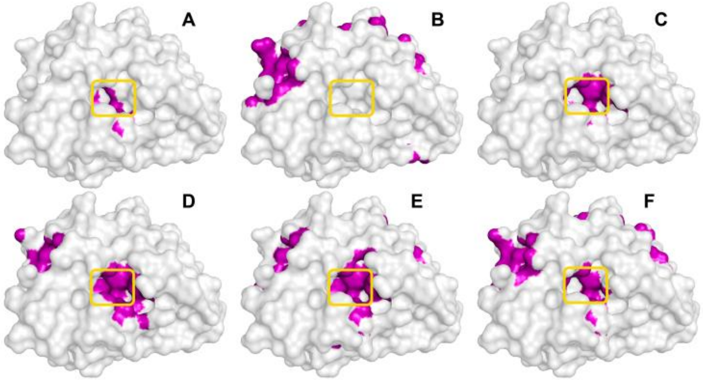

Figure S7. Localization of signaling molecules of fungal QS (γ-butyrolactone (A), butyrolactone I (B),γ-heptalactone (C), multicolanic acid (D), multicolic acid (E) and multicolosic acid (E)) on the surface of **ZEN** enzyme, exhibiting lactonase activity. Molecular surface of enzymes shown as translucent colored gray. The atoms located within 4 Å of any QS molecule atom and the corresponding molecular surface of enzyme, are colored purple. The entrances to the active sites of enzymes are highlighted with yellow boxes.

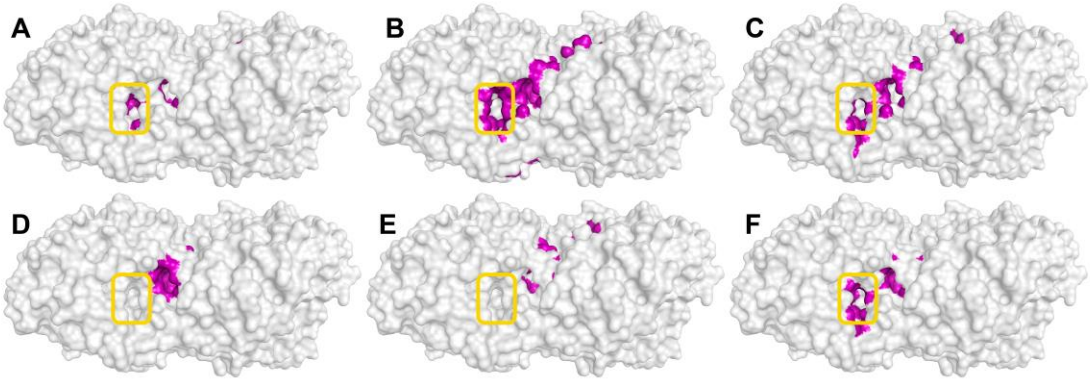

Figure S8. Localization of signaling molecules of fungal QS (γ-butyrolactone (A), butyrolactone I (B),γ-heptalactone (C), multicolanic acid (D), multicolic acid (E) and multicolosic acid (E)) on the surface of **SsoPox** enzyme, exhibiting lactonase activity. Molecular surface of enzymes shown as translucent colored gray. The atoms located within 4 Å of any QS molecule atom and the corresponding molecular surface of enzyme, are colored purple. The entrances to the active sites of enzymes are highlighted with yellow boxes.

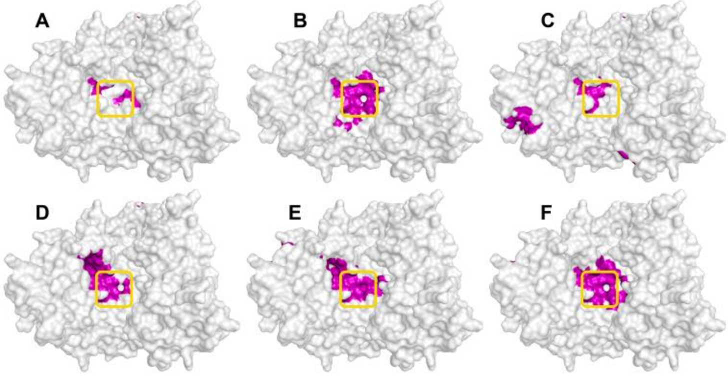

Figure S9. Localization of signaling molecules of fungal QS (γ-butyrolactone (A), butyrolactone I (B),γ-heptalactone (C), multicolanic acid (D), multicolic acid (E) and multicolosic acid (E)) on the surface of **PvdQ** enzyme, exhibiting lactonase activity. Molecular surface of enzymes shown as translucent colored gray. The atoms located within 4 Å of any QS molecule atom and the corresponding molecular surface of enzyme, are colored purple. The entrances to the active sites of enzymes are highlighted with yellow boxes.

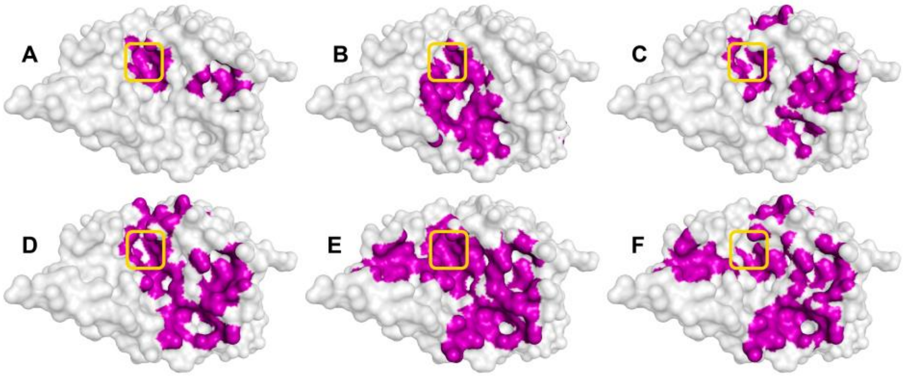

Figure S10. Localization of signaling molecules of fungal QS (γ-butyrolactone (A), butyrolactone I (B),γ-heptalactone (C), multicolanic acid (D), multicolic acid (E) and multicolosic acid (E)) on the surface of **MiM1** enzyme, exhibiting lactonase activity. Molecular surface of enzymes shown as translucent colored gray. The atoms located within 4 Å of any QS molecule atom and the corresponding molecular surface of enzyme, are colored purple. The entrances to the active sites of enzymes are highlighted with yellow boxes.

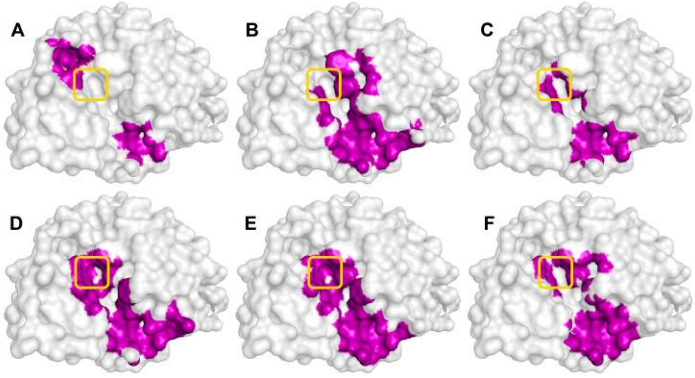

Figure S11. Localization of signaling molecules of fungal QS (γ-butyrolactone (A), butyrolactone I (B),γ-heptalactone (C), multicolanic acid (D), multicolic acid (E) and multicolosic acid (E)) on the surface of **MiM2** enzyme, exhibiting lactonase activity. Molecular surface of enzymes shown as translucent colored gray. The atoms located within 4 Å of any QS molecule atom and the corresponding molecular surface of enzyme, are colored purple. The entrances to the active sites of enzymes are highlighted with yellow boxes.

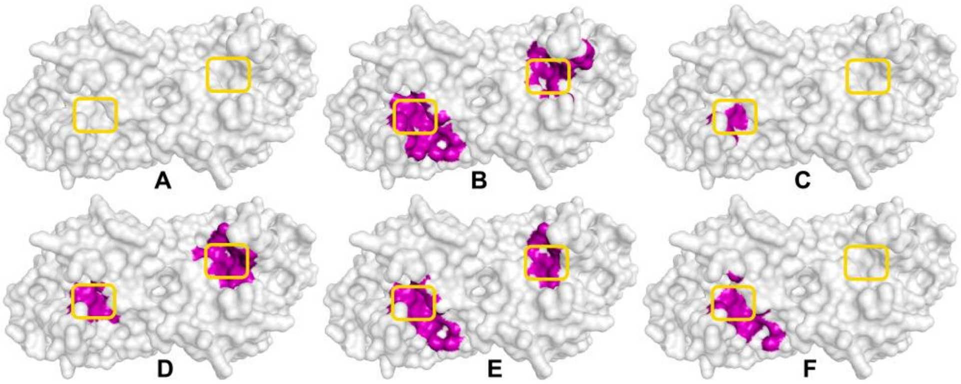

Figure S12. Localization of signaling molecules of fungal QS (γ-butyrolactone (A), butyrolactone I (B),γ-heptalactone (C), multicolanic acid (D), multicolic acid (E) and multicolosic acid (E)) on the surface of **NDM-1** enzyme, exhibiting lactonase activity. Molecular surface of enzymes shown as translucent colored gray. The atoms located within 4 Å of any QS molecule atom and the corresponding molecular surface of enzyme, are colored purple. The entrances to the active sites of enzymes are highlighted with yellow boxes.

A

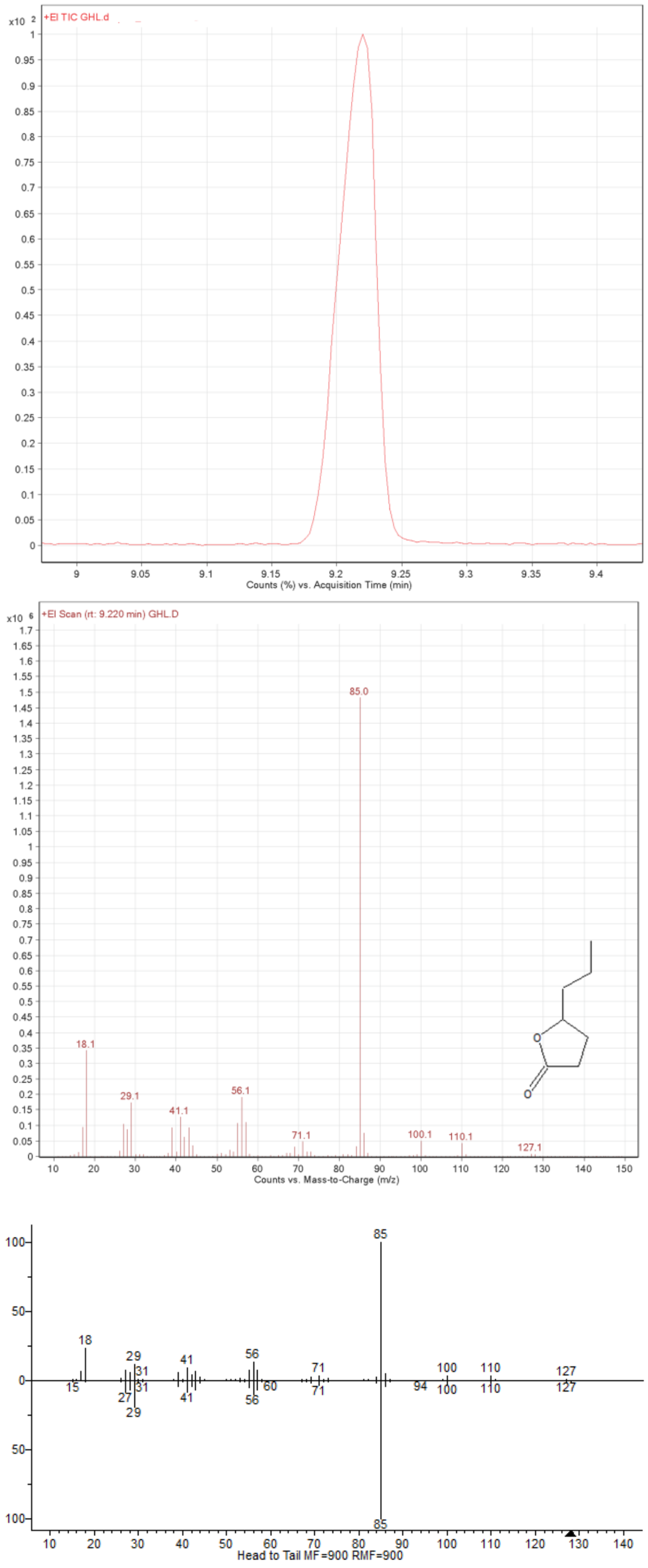

B

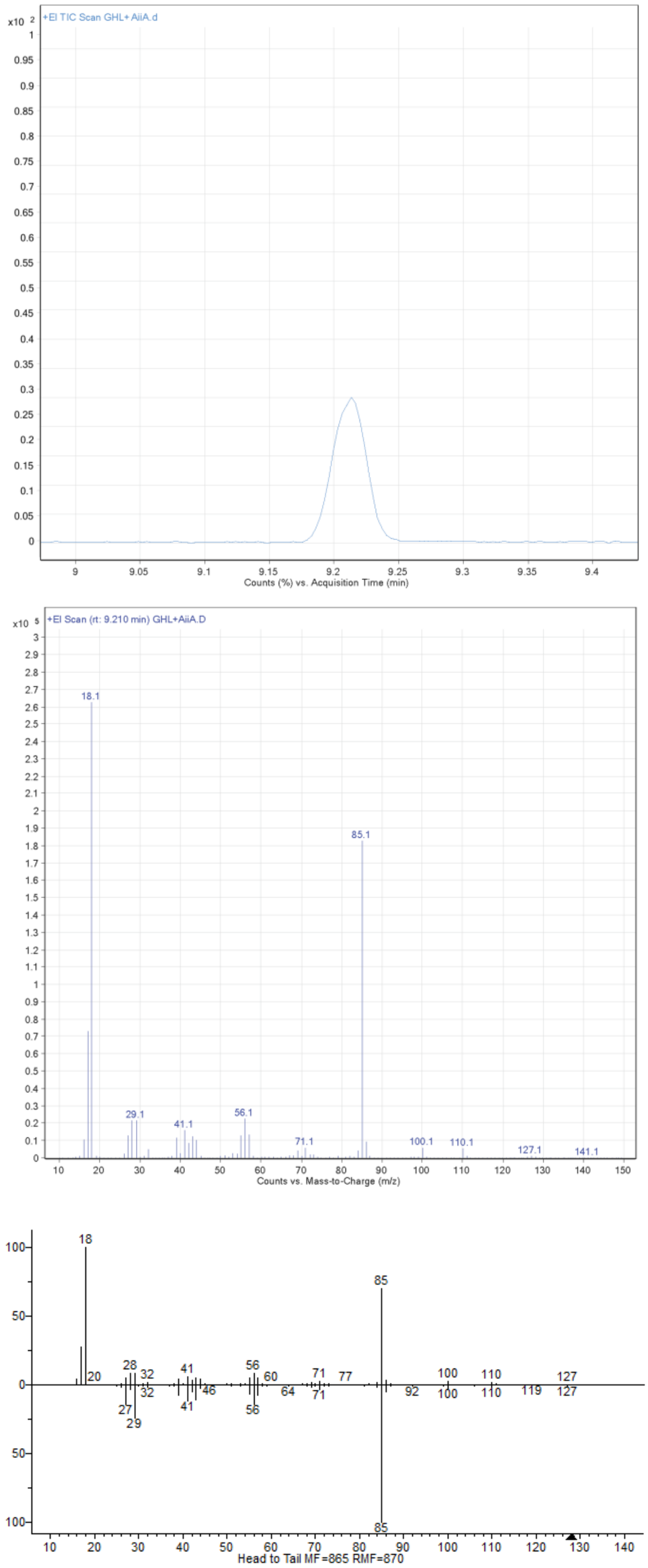
+EI TIC Scan GHL+ AiiA.d
Counts (%) vs. Acquisition Time (min)
+EI Scan (rt: 9.210 min) GHL+AiiA.D
18.1
85.1
29.1
41.1
56.1
71.1
100.1
110.1
127.1
141.1
Counts vs. Mass-to-Charge (m/z)
Head to Tail MF=865 RMF=870

C

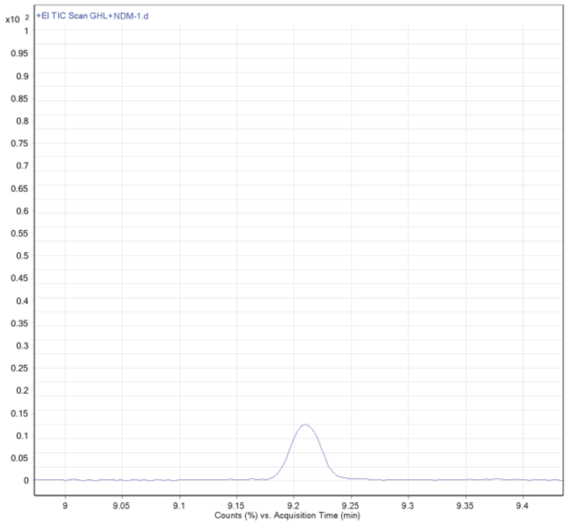

x10 2
+EI TIC Scan GHL+NDM-1.d
Counts (%) vs. Acquisition Time (min)

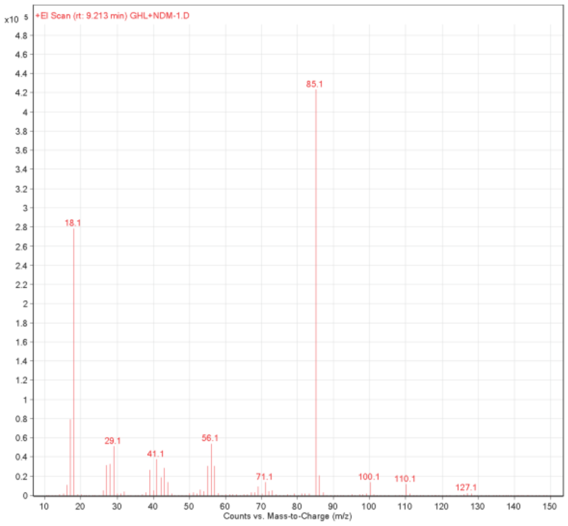

x10 5
+EI Scan (rt: 9.213 min) GHL+NDM-1.D
85.1
18.1
29.1
41.1
56.1
71.1
100.1
110.1
127.1
Counts vs. Mass-to-Charge (m/z)

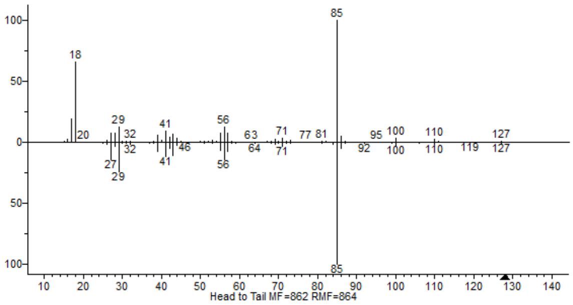

85
18
Head to Tail MF=862 RMF=864

D

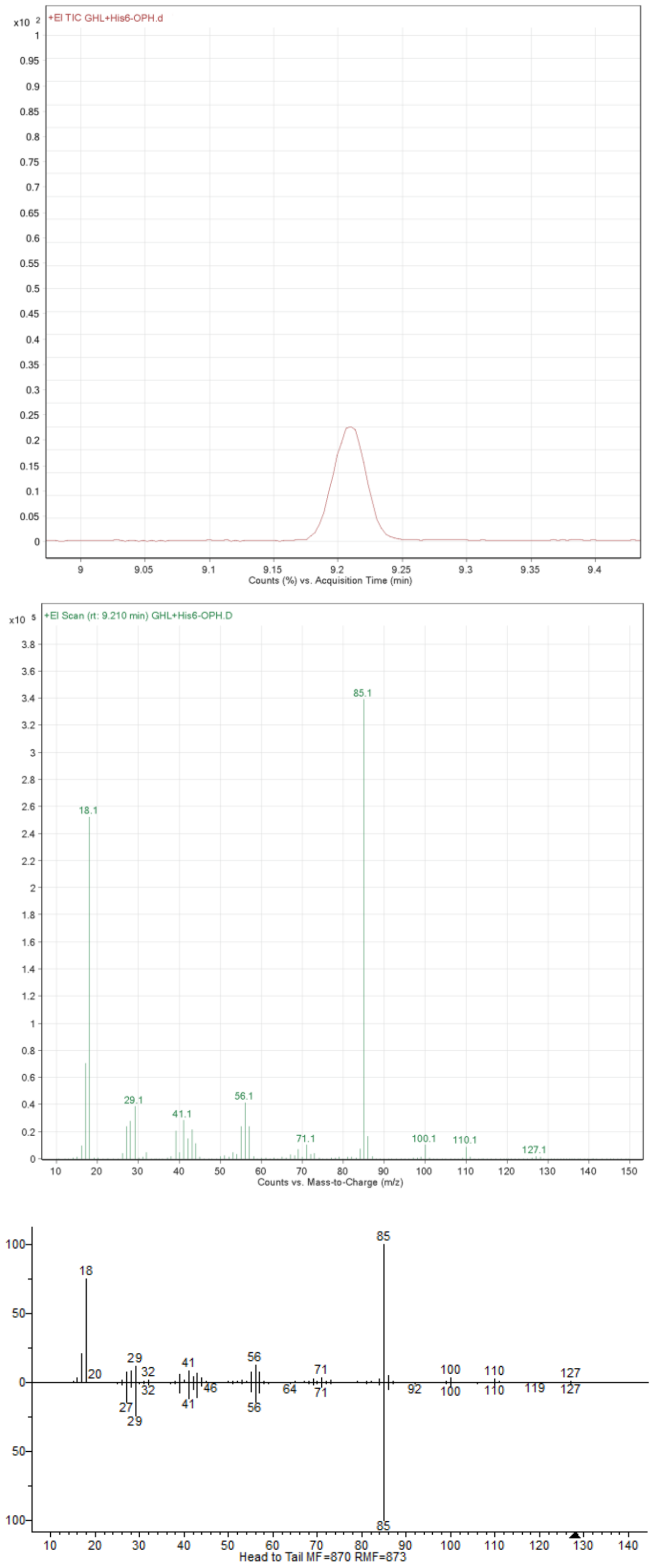

Figure S13. GC/MS analysis of γ-heptalactone (GHL) (**A**) after treatment with enzymes: AiiA (**B**), NDM-1 (**C**) His_6-OPH (**D**). The mass spectra of γ-heptalactone samples peak (top mass spectra) and the mass spectra of γ-heptalactone from the NIST MS library (bottom mass spectra) is also shown. The mass of γ-heptalactone calculated using ChemDraw software and obtained was 128.08 Da and 128.17 Da, respectively.

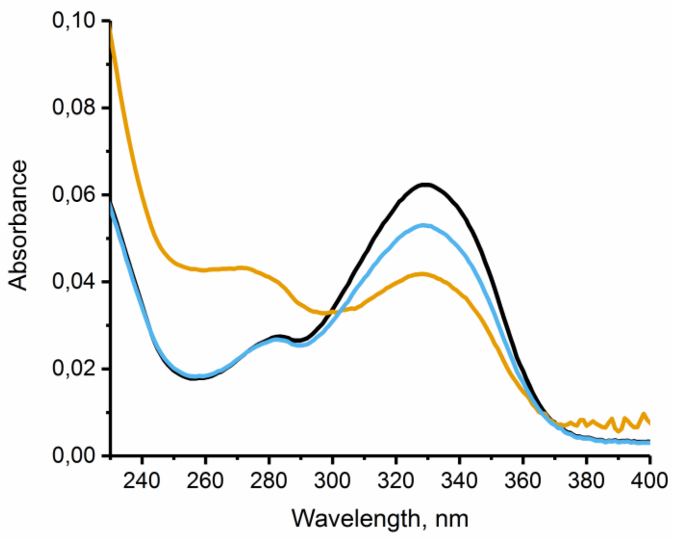

Figure S14. The absorption spectra of butyrolactone I at the initial time (0 h, **black line**) and after 24 h of exposure to natural light in the absence (**blue line**) and the presence (**orange line**) of His_6-OPH.
